# Multiplex Serum Protein Analysis Identifies Novel Biomarkers of Advanced Fibrosis in Patients with Chronic Liver Disease with the Potential to Improve Diagnostic Accuracy of Established Biomarkers

**DOI:** 10.1371/journal.pone.0167001

**Published:** 2016-11-18

**Authors:** Katharine M. Irvine, Leesa F. Wockner, Isabell Hoffmann, Leigh U. Horsfall, Kevin J. Fagan, Veonice Bijin, Bernett Lee, Andrew D. Clouston, Guy Lampe, John E. Connolly, Elizabeth E. Powell

**Affiliations:** 1 Centre for Liver Disease Research, School of Medicine, The University of Queensland, Brisbane, Australia; 2 Statistics Unit, QIMR Berghofer Medical Research Institute, Brisbane, Australia; 3 Department of Gastroenterology and Hepatology, Princess Alexandra Hospital, Brisbane, Australia; 4 Institute of Molecular and Cell Biology, Agency for Science, Technology and Research, Singapore 138673; Singapore Immunology Network, Singapore 138648; and Institute of Biomedical Studies, Baylor University, Waco, Texas, 76798, United States of America; 5 Pathology Queensland, Princess Alexandra Hospital, Brisbane, Australia; Taipei Veterans General Hospital, TAIWAN

## Abstract

**Background and Aims:**

Non-invasive markers of liver fibrosis are urgently required, especially for use in non-specialist settings. The aim of this study was to identify novel serum biomarkers of advanced fibrosis.

**Methods:**

We performed an unbiased screen of 120 serum analytes including cytokines, chemokines and proteases in 70 patients (35 without fibrosis, 35 with cirrhosis on biopsy), and selected a panel of 44 candidate biomarkers, which were subsequently measured in a mixed-etiology cohort of 432 patients with known serum HA, PIIINP and TIMP1 (which comprise the validated Enhanced Liver Fibrosis (ELF) test). Multivariate logistic regression modelling was used to generate models for the prediction of advanced or significant fibrosis (METAVIR ≥F3 and ≥F2, respectively); in addition to identifying biomarkers of disease activity and steatohepatitis.

**Results:**

Seventeen analytes were significantly differentially expressed between patients with no advanced fibrosis and patients with advanced fibrosis, the most significant being hyaluronic acid (HA) and matrix metalloproteinase (MMP) 7 (p = 2.9E-41 and p = 1.0E-26, respectively). The optimal model for the prediction of advanced fibrosis comprised HA, MMP7, MMP1, alphafetoprotein (AFP) and the AST to platelet ratio index (APRI). We demonstrate enhanced diagnostic accuracy (AUROC = 0.938) compared to a model comprising HA, PIIINP and TIMP1 alone (ELF) (AUROC = 0.898, p<0.0001, De Long’s test).

**Conclusions:**

We have identified novel serum biomarkers of advanced liver fibrosis, which have the potential to enhance the diagnostic accuracy of established biomarkers. Our data suggest MMP7 is a valuable indicator of advanced fibrosis and may play a role in liver fibrogenesis.

## Introduction

Liver fibrosis is the main cause of chronic liver disease (CLD)-related morbidity and mortality. The severity of fibrosis, the precursor to cirrhosis, predicts the emergence of complications of portal hypertension and liver-related morbidity and mortality, and therefore influences clinical management. Liver biopsy is the gold standard method for staging hepatic fibrosis; as well as grading inflammatory activity, distinguishing non-alcoholic steatohepatitis (NASH) from fatty liver, and for identifying non-alcoholic fatty liver disease (NAFLD) in patients with other chronic liver diseases. Despite the diagnostic advantages of liver biopsy, the procedure is invasive, costly, requires specialised expertise; and is also limited by its semi-quantitative nature, sampling error and intra-observer variability[1]. With the growing prevalence of CLD, especially NAFLD, and the introduction of highly efficacious direct acting antiviral (DAA) agents for HCV, there is an increasing need for non-invasive biomarkers to stratify risk and assess disease progression/regression; to facilitate larger scale screening and provide efficacy endpoints for clinical trials.

A number of non-invasive methods of fibrosis assessment have been identified and widely validated, including imaging techniques and serum biomarkers. The use of non-invasive biomarkers for excluding advanced fibrosis to reduce the number of liver biopsies has been incorporated into clinical practice guidelines, however they are still considered insufficiently accurate for assessing intermediate stages of fibrosis, disease progression or the effect of therapy[1]. Transient elastography, which can reliably identify advanced fibrosis, is one of the most frequently used non-invasive methods; however its use is largely limited to specialist centres due to the need for specialised instrumentation and expertise. Serum biomarkers are more suitable for use in general clinical practice, and several serum tests have been developed using combinations of direct (associated with liver fibrogenesis) and/or indirect (reflecting liver function) biomarkers. Serum panels offer several advantages over liver biopsy, as they are quantitative and have the potential to reflect the dynamic nature of fibrogenesis, providing a more sensitive assessment of the dynamic changes associated with fibrosis progression/regression compared to static fibrosis stage. Complex panels incorporating direct markers, such as the Enhanced Liver Fibrosis (ELF) test[2], FibroTest[3] and Hepascore[4], are generally thought to be superior to simple panels such as the aspartate aminotransferase (AST) to platelet ratio index (APRI). The available complex panels perform similarly for the detection of advanced fibrosis[5, 6], with reported area under the receiver-operating curve (AUROC) values of 0.8–0.9. However, a recent meta-analysis of nine studies evaluating the ELF test reported median sensitivity/specificity values of 78%/76% for advanced fibrosis, although the data-driven diagnostic cut-offs applied in different studies were a major cause of heterogeneity[7]. Using the ELF manufacturer’s cut-off for advanced fibrosis (≥9.8), we demonstrated 92% specificity and 74% sensitivity for the detection of advanced fibrosis in a mixed etiology CLD cohort[8].

The identification of serum biomarkers initially focussed on candidate approaches, assessing serum levels of proteins/peptides implicated in liver fibrogenesis, in particular matrix remodelling. More recently, unbiased approaches have been undertaken, including protein[9] and metabolite[10] profiling, with the goal of discovering novel biomarkers that improve the diagnostic accuracy of the currently available tests and provide new insights into mechanisms of disease pathogenesis. The aim of this study was to identify novel serum biomarkers of advanced liver fibrosis (METAVIR fibrosis stage 3 or 4 on liver biopsy) using multiplex ELISA-profiling, including cytokines, chemokines, and matrix remodelling factors, in a mixed etiology cohort of CLD patients with known ELF scores[8]; and to evaluate their diagnostic accuracy using liver biopsy as the reference. The underlying rationale for this approach was that, since fibrogenesis involves activation of many liver cell types in response to injury, factors reflecting different pathological processes (eg hepatitis/inflammation or metabolic perturbations, in addition to matrix remodelling) could provide an optimal biomarker panel.

## Patients and Methods

### Patients

This is a retrospective, cross-sectional study in 432 patients referred to the Princess Alexandra Hospital hepatology outpatient clinic between 1999 and 2013 for CLD management who underwent a liver biopsy. The cohort is the sub-set of a previously reported cohort of 536 consecutive patients[8] for whom sufficient, non-hemolyzed serum remained for multiplex ELISA analysis. Fasted serum collected at the time of liver biopsy was stored at -80°C prior to analysis. Informed written consent was obtained from each patient and the protocol was approved by the Metro South Health and The University of Queensland Human Research Ethics Committees. The study protocol conforms to the ethical guidelines of the 1975 Declaration of Helsinki as reflected in a priori approval by the Institution's human research committee. Diagnosis of liver disease was based on standard biochemical and serological assays and consensus histological assessment of the liver biopsy by 2 blinded, experienced pathologists (ADC and GL) as previously reported [8]. Eighty-five % of liver biopsies were ≥15 mm in length or showed definite cirrhosis (98% of biopsies were ≥10 mm). Fibrosis was assessed using a modified METAVIR score[11] as follows: stage 1, portal or central fibrosis; stage 2, some septa; stage 3, many septa; stage 4, cirrhosis. The METAVIR scoring system was used to assess hepatic inflammatory activity[11]. Steatosis was defined as >5% steatotic hepatocytes. Steatohepatitis was identified by the presence of steatosis, inflammation and ballooned hepatocytes. Weight, height, age, serum biochemistry (AST, ALT) and platelet counts were obtained from the medical record and used to calculate the AST to Platelet Ratio Index (APRI)[1] and Fibrosis-4 score (FIB4)[1]. An ADVIA Centaur XP system (Siemens Healthcare Diagnostics, New York, USA) was used to measure serum HA, PIIINP and TIMP1 and calculate the ELF score [8].

### Multiplex ELISA

Serum analytes were measured using 10 human luminex panels (Merck, Darmstadt, Germany): Cytokine-1, Cytokine-2, Cytokine-3, Adipokine-1, Adipokine-2, Liver, Metabolic Hormone, MMP-1, MMP-2, and Soluble Cytokine Receptors. Analyte selection was guided by panel availability, cross-vendor quality control and based on an analysis of an internal reference data set of 680 normal donors. As this was a discovery set, our aim was to interrogate as broad a panel as possible focusing on growth factors and immune effectors, e.g. cytokines, chemokines and proteases. Dilutions were as recommended by the manufacturer. Plates were read and analysed using the Flexmap 3D (Luminex Corp) and Bio-plex Manager software (Bio-Rad). Data outside the range of the standards, but within the asymptote of the equation, were extrapolated beyond the standard curve. Candidate biomarkers for the focussed screen were chosen from the pilot data (Table A in S1 File) by selecting the 5 Luminex panels that contained (a) the analytes most significantly associated with fibrosis, followed by (b) the greatest number of analytes significantly associated with fibrosis or other histopathological features. All fibrosis-associated analytes from these 5 panels were included, along with selected analytes that were associated with activity or steatohepatitis.

### Statistical methods

Patient characteristics were summarized by mean and standard deviation for continuous variables and frequency and percent for categorical variables. Exploratory univariate analyses were performed using t-tests with Bonferroni-Holm multiple testing correction to compare serum analyte levels in patients with different histological features on biopsy; namely fibrosis stage, activity, steatosis and steatohepatitis (Tables A and B in S1 File).

### Variable selection and multiple logistic regression modelling

Analytes with >20% missing values were excluded from the analysis (IL10, IL6, IL7, LTA, CCL26, IL20, IL23, IL33, TSLP, FGF21, FGF23). For the remaining analytes, missing values due to out of range measurements were imputed using the maximum or minimum detectable value. The 17 missing BMI values were imputed by median imputation. After exclusion of missing data, 33 luminex analytes, HA, PIIINP, TIMP1 and four clinical covariates that were significantly associated with METAVIR stage (age, diabetes, BMI and APRI) from 430 patients were used to create prediction models for advanced fibrosis (METAVIR ≥F3), significant fibrosis (METAVIR ≥F2), moderate-severe activity (METAVIR grade 2–3) and steatohepatitis. Gender frequency did not differ according to METAVIR Stage. Serum analytes and APRI were log transformed.

The variables for the multivariable logistic regression model were selected by lasso[12]. The shrinkage parameter, lambda, was chosen by taking an average of the estimated lambdas that minimized the mean 10-fold cross–validation error in 50 randomly created subsamples[13]. The variables with non-zero coefficients resulting from the lasso were used for multivariable logistic regression. Only variables that were significant (p<0.05) in the multivariable logistic regression were retained in the final model. Due to the relatively small number of subjects with advanced fibrosis (n = 90), splitting the data into training (n = 60) and test (n = 30) sets resulted in unstable estimates of prediction performance that were highly dependent on test set selection. As such, model generation and testing was performed using the 430 patients. The results of the final model were presented as Odds Ratios with 95% confidence intervals and p-values. To prevent an unfair comparison between models, an “ELF model” was re-built on the 430 patients from serum HA, PIIINP and TIMP1. Prediction performance and model comparisons were assessed by evaluating sensitivity, specificity, positive and negative predictive values and AUROC values (with comparison using DeLong’s test). Statistical analysis was performed in R v3.2.2.

## Results

### Study Population

Fasting serum was obtained from a mixed-etiology cohort of 432 patients at the time of liver biopsy at a single centre. The majority had chronic HCV infection (266 (62%)) and 92 (21.3%) had advanced fibrosis (METAVIR stage F3-F4) (Table 1).

**Table 1:** Patient characteristics.

	F0	F1	F2	F3	F4	All
Age	38.4 (10.3)	41 (10.3)	44.2 (9.8)	47.8 (11.3)	50.4 (9.5)	43.1 (10.8)
mean (sd)						
Gender	22 (44)	115 (61)	69 (68)	34 (69)	37 (86)	277 (64)
Male, n (%)						
BMI*	25.6 (4.3)	26.3 (5.4)	27.1 (5.8)	25.9 (4.6)	28.4 (5.1)	26.6 (5.3)
mean (sd)						
ALT	83.7	92.5	118.9	159.3	133.3	109.8
Mean (sd)	(68.2)	(86.1)	(88.5)	(170.2)	(121.8)	(104.8)
AST	46.9	54.3	75.7	106.5	91.04	68.5
Mean (sd)	(28.2)	(39.9)	(52.2)	(99.02)	(64.12)	(57.8)
HCV	26	123	66	26	25	266
HBV	7	32	16	14	9	78
NAFLD	9	18	8	4	7	46
ALD	3	4	4	3	1	15
Other#	5	11	8	2	1	27
**Total**	50	188	102	49	43	432

*17 missing

#Other: immune-mediated n = 14, HFE hemochromatosis n = 3, diabetes/glycogenesis n = 3, erythrocytic erythropoietic protoporphyria n = 1, granulomatous hepatitis n = 2, methotrexate n = 1, previous HBV n = 1, veno-occlusive disease n = 1, non-specific chronic hepatitis n = 1.

### Identification of serum analytes associated with advanced fibrosis

**Fig 1:**
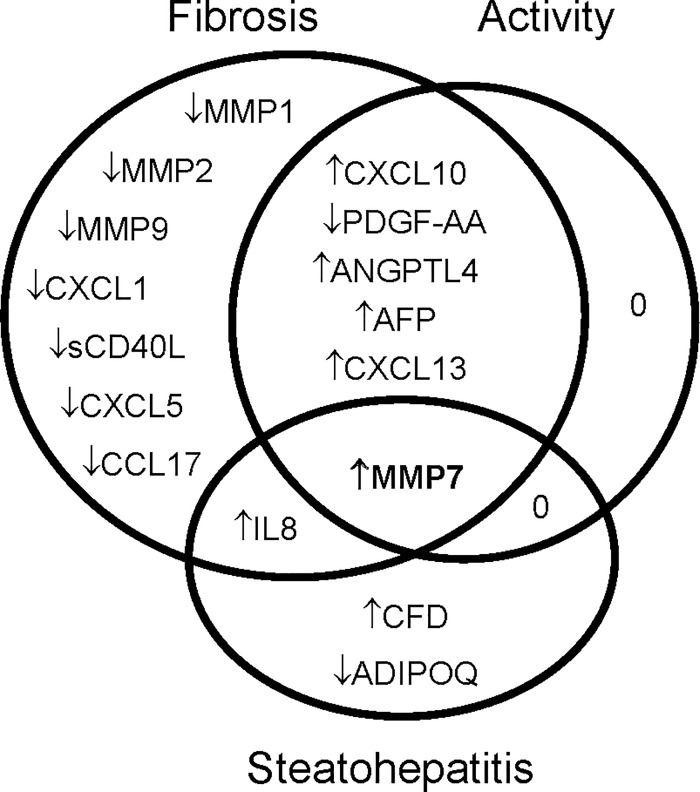
Serum proteins associated with advanced fibrosis (>F2), moderate-severe activity (METAVIR Grade 2–3) and/or steatohepatitis (Bonferroni-Holm corrected p<0.05).

A broad screen measuring 120 unique serum analytes, including chemokines, cytokines and matrix remodelling enzymes (10 multiplex ELISA panels comprising a total of 132 analytes), was conducted in a randomly selected sub-cohort of 35 patients without fibrosis (F0) and 35 patients with cirrhosis (F4); with a similar spectrum of etiologies to the whole cohort. Seventy-six percent (101) of the selected analytes were measurable in serum from ≥90% patients. Thirty-one analytes were significantly different in serum from cirrhotic patients compared to patients with no fibrosis (p<0.05, Table A in S1 File). Multiple analytes showed significant associations with other pathological features of CLD, including inflammation (METAVIR activity grade), steatosis and steatohepatitis, as well as, or in addition to, fibrosis (Table A in S1 File). Forty-four candidate biomarkers that were significantly associated with fibrosis and/or other pathological features of CLD by univariate analysis were selected for further analysis using the full cohort of 432 sera (Fig 1, Table B in S1 File).

### Variable Selection and logistic regression models for predicting advanced liver fibrosis (F3-F4)

**Fig 2:**
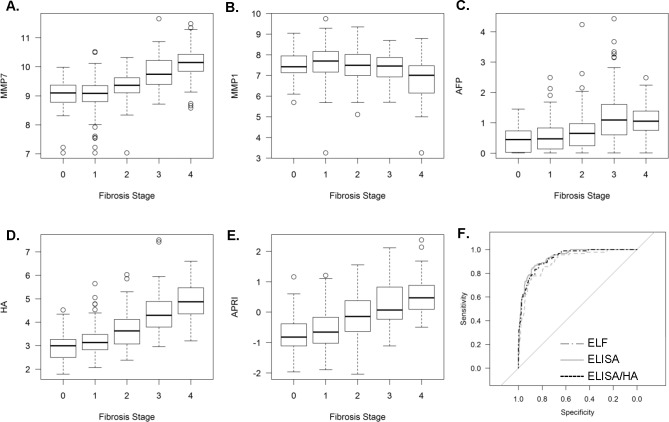
Components of Logistic Regression Model for Predicting Advanced Fibrosis. Serum MMP7 (A), MMP1 (B), AFP (C), HA (D) and the APRI (E) were selected in the optimal linear regression model for advanced fibrosis (ELISA/HA model). (F) AUROCs for the ELISA, ELF/HA and ELF models.

Variable selection and logistic regression models were generated and evaluated on the whole sample of 430 patients. The optimum multivariable prediction model from the 33 analytes remaining after controlling for missing data and 4 clinical co-variates (age, BMI, APRI and diabetes), incorporated MMP7, MMP1, AFP, PDGF-AA, APRI and age (Table 2, AUROC = 0.928, “ELISA model”). When TIMP1, HA and PIIINP, which are established biomarkers of fibrosis used in the ELF test, were included in the variable selection step the multivariate model included MMP7, MMP1, AFP, HA and APRI (AUROC = 0.938, Table 2, Fig 2, “ELISA/HA model”).

**Table 2:** Multivariate logistic regression models for predicting advanced fibrosis. Variables selected by lasso from 33 serum analytes measured by multiplex ELISA and clinical covariates excluding (Model 1) or including (Model 2) the ELF test components (HA, PIIINP and TIMP1) were used to generate multivariate logistic regression models for advanced fibrosis (METAVIR >F2).

	Model 1: ELISA		Model 2: ELISA/HA	
Variable	Odds Ratio [95% CI]	p-value	Odds Ratio [95% CI]	p-value
HA	[not available]		1.17 [1.12–1.22]	<0.0001
MMP7	1.2 [1.15–1.27]	<0.0001	1.15 [1.10–1.21]	<0.0001
APRI	1.12 [1.08–1.18]	<0.0001	1.06 [1.01–1.11]	0.019
AFP	1.09 [1.04–1.15]	0.0004	1.06 [1.01–1.12]	0.016
MMP1	0.93 [0.90–0.97]	0.0004	0.93 [0.90–0.96]	0.0001
Age	1.01 [1.00–1.01]	0.0003	not selected	n/a
PDGF-AA	0.93 [0.88–0.99	0.0232	not selected	n/a

In order to compare the performance of our new models, we used HA, PIIINP and TIMP1 to generate an ELF model on the same data set. Table 3 shows the sensitivities, positive and negative predictive values (for specificity 80%) and AUROC values for the newly-generated ELF model and the models incorporating the novel analytes that were identified. The ELISA/HA model afforded the highest sensitivity (88.9%), followed by the ELISA model (86.7%) and the ELF model (81.1%). The AUROC of the ELISA/HA model was significantly higher than that of the ELF model, whilst improved performance of the ELISA model approached significance (p<0.001 and p = 0.054 respectively, DeLong’s test, Table 3, Fig 2). Both the ELISA/HA and ELF models classified the majority of patients to the same fibrosis stage (Table 4), with 71.1% of F0-2 patients and 80.0% of F3-4 patients correctly classified, 52 (20%) F0-2 patients classified as advanced and 9 (10.0%) F3-4 patients classified as non-advanced by both scores. However, reflecting the difference in model sensitivity, ELF incorrectly classified an additional 8 (8.9%) stage 3–4 patients, whereas the new model resulted in only 1 (1.1%) additional false negative. As previously shown for the ELF score[8], false positive results from the ELISA model were significantly associated with increased age and disease activity (data not shown).

**Table 3:** Performance of the ELF, ELISA and ELISA/HA models for the prediction of advanced liver fibrosis (METAVIR >F2).

	ELF Model	ELISA Model	ELISA/HA Model
**Sensitivity**	0.811	0.867	0.889
**specificity**	0.8	0.8	0.8
**PPV**	0.518	0.534	0.541
**NPV**	0.941	0.958	0.965
**AUROC**	0.898	0.928	0.938
P value*		0.054	<0.001

*compared to ELF Model

**Table 4:** Comparison of patient classification by the ELF and ELISA/HA models. Patients with non-advanced (F0-2) or advanced (F3-4) fibrosis on biopsy were diagnosed by the ELF or ELF/HA model (specificity 80%). True negative and true positive patients identified by both tests are shaded grey; concordant and discordant misclassification rates for the 2 models are shown.

**F0-2 Patients**		**ELISA/HA**	
		F0-2	F3-4
**ELF**	F0-2	**256 71.1%**	16 4.4%
	F3-4	16 4.4%	52 20.0%
**F3-4 Patients**		**ELISA/HA**	
		F0-2	F3-4
**ELF**	F0-2	9 10.0%	8 8.9%
	F3-4	1 1.10%	**72 80.0%**

### Variable Selection and logistic regression models for predicting significant liver fibrosis (F2-F4)

The optimum multivariable prediction model for predicting significant fibrosis from the 33 analytes measured by ELISA and 4 clinical co-variates incorporated age, IL8, MMP7, MMP1 and APRI (Table C1 in S1 File, AUROC = 0.865). When the components of the ELF score were included in the variable selection step, the model included MMP7, TARC, TIMP1, HA and APRI (Table C2 in S1 File, AUROC = 0.873). Similar to the advanced fibrosis models, the addition of novel analytes significantly improved the diagnostic accuracy of the ELF model (Table C3 in S1 File p = 0.0021).

### Variable Selection and logistic regression models for predicting disease activity and steatohepatitis

Whilst our study was designed to identify biomarkers of advanced fibrosis, our panel also included putative biomarkers of liver inflammation and steatohepatitis. In both the pilot and focused screens there was, not surprisingly, considerable overlap between analytes that were significantly associated with fibrosis stage and activity grade or steatohepatitis (Fig 1, Tables A and B in S1 File). To identify the best biomarkers of inflammation and steatohepatitis among the measured analytes we performed variable selection logistic regression. The best model for predicting liver inflammation (METAVIR grade 2–3) comprised CXCL10, TIMP1 and APRI (AUROC = 0.798, Table D in S1 File); whereas the best model for predicting steatohepatitis incorporated IL8, ADIPOQ, MMP2, MMP7, age, BMI and diabetes (AUROC = 0.857, Table E in S1 File).

## Discussion

The extent of liver fibrosis is the most important predictor of liver-related outcomes, including development of portal hypertension, decompensation events, cancer and death. Although the limitations of liver biopsy itself have prompted suggestions that clinical outcomes be used as the reference standard, biopsy remains the gold standard as the cohort sizes and length of follow-up required would be impractical. The few studies to date do, however, indicate that serum biomarkers perform similarly to histology for predicting clinical outcomes[14–16]. Overcoming the diagnostic limitations of liver biopsy, against which novel biomarkers are benchmarked, is likely a key obstacle to refining non-invasive measures—it has been estimated that even in the best possible scenario an AUROC>0.90 could not be achieved for a perfect marker of fibrosis[17]. Furthermore, liver biopsy, as with all non-invasive modalities investigated to date, also suffers from high rates of misclassification of intermediate and adjacent disease stages[18].

Currently recommended as an adjunct tool to liver biopsy, the primary clinical utility of non-invasive biomarkers lies in their ability to reliably rule out advanced fibrosis. Non-invasive serum tests, including indirect non-expensive algorithms, perform reasonably well in this regard, achieving AUROCs of ~0.8. However, given the size of the population at risk of CLD, the increasing demands on hepatology services and the implications of failing to identify advanced fibrosis, striving to maximise the diagnostic accuracy of non-invasive tests is an important goal. In this study we identify serum MMP7, MMP1 and AFP as biomarkers of advanced fibrosis, which, when combined with HA and the APRI, generated a model with excellent diagnostic accuracy (AUROC 0.938), which was superior to a model comprising the ELF test analytes alone. Our new model has the potential to increase sensitivity by 5–10% for the same specificity. Maximal sensitivity is particularly important for a tool that may be used in a screening capacity where disease prevalence is relatively low, in order to reliably exclude advanced fibrosis. Given the low prevalence of advanced fibrosis in the general population, simple, non-expensive strategies to identify high-risk subjects for further testing could also be used to improve the positive predictive value of the more complex panels[19].

MMP7, MMP1, HA and the APRI were consistently selected in the models for significant and advanced fibrosis. Hyaluronic acid, a liver extracellular matrix component produced by hepatic stellate cells, is a well-established biomarker of advanced liver fibrosis, which is utilised in several non-invasive tests, including ELF, Hepascore and Fibrometer. Common clinical/demographic parameters are also routinely incorporated in non-invasive tests, including platelets, liver enzymes, BMI, bilirubin and ferritin[1]. Our results support the utility of APRI, one of the simplest and best-validated predictors of advanced fibrosis, in complex panels. It is not surprising that the matrix remodeling enzymes MMP7 and MMP1, along with MMP2 and MMP9, were strongly associated with fibrosis; as matrix remodeling factors have long been assessed in candidate biomarker identification approaches (although MMP7 is little studied in the liver). We hypothesise that these, and other biomarkers incorporated into our models may provide new functional insights into liver fibrogenesis. Given this is the most comprehensive, unbiased serum protein screen in CLD of which we are aware, it was interesting, and perhaps surprising, that few chemokines and cytokines were selected as biomarkers, although our screen included known liver injury and inflammation-associated molecules. Cytokines and chemokines such as CXCL10, IL-8 and Adiponectin were, however, selected in models predicting histological inflammation and NASH (although the study was not designed to identify optimal biomarkers of these features). IL-8 is a particularly interesting candidate biomarker, which has previously been observed to be elevated in patients with NASH, given its role in neutrophil recruitment and association with hepatocyte senescence, both of which are increasingly implicated in NASH pathogenesis[20–22].

The most significant and novel biomarker for advanced liver fibrosis we discovered was MMP7, which had a similar odds ratio to HA for the prediction of significant or advanced fibrosis. MMP7 has been reported as a promising biomarker in various malignancies, including liver cancer, and pulmonary fibrosis[23, 24], and an automated assay suitable for routine clinical use has recently been developed. In the liver, MMP7 is expressed by hepatocytes, biliary epithelial cells and Kupffer cells[25], and increased MMP7 expression has been associated with cirrhosis[26], biliary atresia-associated fibrosis[25] and HCC migration and invasion[27]. Together, these studies suggest MMP7 may be a novel player in the pathogenesis of CLD, and a candidate ‘direct’ biomarker reflecting dynamic fibrogenesis. MMP1, the major fibrillary collagenase, has previously been identified as a fibrosis biomarker—it is a component of the MP3 fibrosis score (MMP1 and PIIINP). In contrast to the increase in MMP7, advanced fibrosis was associated with reduced serum MMP1, which may suggest a perturbation in the balance between matrix deposition and degradation contributes to progressive fibrosis. In support of this, MMP1 levels were previously reported to be elevated in patients with mild fibrosis compared to healthy controls, but diminished in cirrhosis[26], and we recently demonstrated exacerbated fibrosis in mice deficient in macrophage Wnt production which was associated with reduced Mmp13 expression (the murine ortholog of MMP1)[28]. AFP, a hepatoblast marker, is used clinically as a biomarker for HCC, although an association with fibrosis has previously been observed in chronic HBV[29], and reduced AFP levels were associated with fibrosis regression following interferon therapy in patients with chronic HCV[30]. Given the alternative, hepatic progenitor cell (HPC)-mediated pathway of liver repair is increasingly activated during CLD progression, and immature HPC are the source of AFP[31]; AFP could represent a novel serum biomarker of HPC activation.

There are several limitations to this study. Patients were recruited from a tertiary centre and there was a preponderance of HCV and F1-2 patients, which could introduce selection and spectrum biases, respectively. The performance of our models may be over-estimated in absolute terms, since they were generated and evaluated on the same cohort, however we generated a new ELF model in order to compare the relative performance of the models. This study included a mixed-etiology cohort, dominated by subjects with HCV; and although the performance of serum biomarkers has been shown to differ according to etiology we did not have sufficient power to investigate this. Finally, liver specificity is a potential issue with serum biomarkers in general; HA, for example, can be affected by liver or renal failure, and MMP7 is associated with cancers and other fibrotic conditions.

In conclusion, we report the most comprehensive, unbiased screen for serum analytes associated with advanced liver fibrosis. With increasing demand for specialist hepatology services and a large proportion of the population at risk of CLD, the development of sensitive biomarkers for fibrosis is an important goal. We identify a novel, highly sensitive panel of candidate biomarkers for advanced fibrosis. Our results indicate that MMP7, in particular, is strongly associated with advanced fibrosis and may represent a valuable serum biomarker and novel player in CLD pathogenesis.

## Supporting Information

S1 File **Table A.** Associations of 132 serum analytes measured in 70 patients with Chronic Liver Disease (35xF4, 35xF0) with liver fibrosis, activity, steatohepatitis, steatosis and ELF score. Differential analyte expression with histological features was analysed by t-test with Bonferroni-Holm correction, and correlation with ELF score was analysed by spearman correlation. **Table B.** Associations of 44 serum analytes measured in 432 patients with Chronic Liver Disease with liver fibrosis, activity, steatohepatitis, steatosis and ELF score. Differential analyte expression with histological features was analysed by t-test with Bonferroni-Holm correction, and correlation with ELF score was analysed by spearman correlation. **Table C.** ELISA Model for the prediction of significant fibrosis (METAVIR F2-4) **Table D.** Multivariate logistic regression model for the prediction of moderate-severe disease activity (METAVIR Grade 2–3) (n = 430 patients). **Table E.** Multivariate logistic regression model for the prediction of steatohepatitis (n = 400 patients).
(XLSX)
